# What Is Vitality of a Tooth?

**Published:** 1883-07

**Authors:** Charles Mayr


ARTICLE V.

### WHAT IS VITALITY OF A TOOTH?

BY CHARLES MAYR, SPRINGFIELD, MASS.

We often hear the expressions, "lowered vitality," "reduced vitality," "great vitality," What exact meaning and sense can we give to those words? To understand our explanation of those terms, one has to accept our explanation of what vital force is: *Vital force in a tissue is the result of all the forces active in that tissue of whose particular vital force we speak.* Thus, e. g., the vital force in the stomach is the resultant of the forces in the following components; the gross outer structure, the three different layers, the gravity, the circulation, the quality of the blood.

Those are the grossly mechanical outer factors. Then comes the, so to speak, microscopical factors, viz: The structure of the three layers, the arrangment of the different glands, the structure of those glands, the nature of the recticulum, and finally the size and quality of the recticulum.

Each of those factors is again a compound of many smaller factors, and thus our vital force in the stomach becomes an extremely complicated total.

Let us take the tooth. What is the vital force in a tooth? It is the resultant of all the forces therein. The very vitality of a tooth may prove its de tructive element. The following factors of the vital force in a tooth are important to us: Its chemical composition, its mechanical arrangement, its circulation. (About the first we know in detail only a little to a certain point.) We know the proportion of lime-salt and organic substance in dried teeth, etc. The vitality of a tooth may be said to depend on those three factors, and changes in them will produce certain changes in the "vitality" of a tooth, viz: Changes in the composition, changes in the mechanical structure, and finally charges in its circulation. The last is the most important factor of all the changes. The first two factors are more what we may term congenital factors, while the last depends on other circumstances. We may, therefore, for a moment neglect changes in composition and structure and chiefly consider the changes of the circulation, which is most likely to vary. We can no longer doubt that the most important element in the composition of a tooth is the organic albuminous protoplasm; called thus far as chemical processes are concerned,—or called bioplasson as far as its structure and the mechanical processes in it are being considered. The nature of this bioplasson can probably not alter much in its mechanical arrangement this being given by the canaliculi of the tooth, but its composition may change. If we have two persons, say both weighing 180 pounds, the one a flabby, "fleshy" woman, the other a strong, healthy man, what is the difference? The bones? Not so much—hardly two pounds more in one case than in the other. The difference is in the water and fat; both are inert as far as the real working and superintending of the system is concerned; all the brain work of the system is done in the last instance by the protoplasmatic elements of tissue, and there is where the difference comes in. The fat woman is made up of inert water and fat, while the healthy man shows a larger percentage of albuminous elements. The general structure of the reticulum in both bodies is very nearly the same, but the matter compounding it is stronger in the latter case than in the former, because of its grater concentration. Suppose the thigh muscles of both parties to be weighed, and both be found to weigh the same amount. the muscles in the first person will be composed of 90 per cent. fat and water, and 10 per cent. albumen; in the second person, of 75 per cent. fat and water, and 25 per cent albumen. The second person will be two and one-half times stronger than the first because he has two and one-half times more organic albuminous substance, because the threads of the reticulum in the muscles probably owe their strength to the abuminous matter alone, and but little to the water and fat. This will illustrate what we understand by vitality of teeth. What we said the fat woman and the strong man is only an illustration, and an analogy of very close resemblance because of its dealing with great vitality and teeth with low vitality? The first are teeth whose reticulum contains relatively little water and inert substances, while the second are teeth whose reticulum of bioplasson contains very much water. As albumen, however, has nearly the specific gravity of water, the weight of a tooth will not be changed very much by such a change in the composition, and the gross analysis for the lime-salts alone is not affected at all by those changes. *The proportion of water to protoplasm in a tooth is a very sure measure of its "vitality."* We have the more "vitalty" the greater the percentage of albuminous substance to water. From this standpoint we can explain the lowering of vitality of teeth in consequence of diseases that affect the rest of the body. During all diseases we live more or less on our own meat. The brain is the last organ—the chief of the body—that will show a loss; all the other organs are eaten up in supplying the brain, and just as the muscles of a starving person shrink because the brain eats them up, thus also the bioplasson shreds of a tooth will shrink because the brain will eat it up; but the shrinkage will not bring in its course a diminution of the volume of the tooth. The structure does not allow of that very much; the vacuum will be filled out with water. Starving persons, as it is well known, drink large quantities of water, and if they are allowed an unlimited supply of water they are able to stand hunger many days longer than when deprived of it. While the albuminous and fatty contents of the tissues are consumed, water is taken into their place. Thus also with the teeth. The teeth become richer in water but poorer in protoplasm.

Anything, therefore, which leads to consumption of protoplasm in the body, be it dropsy or consumption or pregnancy, must necessarily draw protoplasm from other tissues, where it is not absolutlly needed. But the outside enemy has not got the consumption or pregnancy or any of that kind. While, therefore, the system is weakening its fortifications, the enemy does not weaken, and, as a consequence, whenever there is a chance, that enemy will crowd in. If we see the forces of the attacking micro-organisms and those of the protoplasma within a tooth exactly balanced with a protoplasma of a strength, say of 20 per cent., of solid substance, and if by some disease the percentage has fallen to 10 per cent., the interior resistance has fallen to one-half, and hence the increased liability to diseases by the relative change of forces. We do not enter into the still more remote detail of the "How" of the action of the protoplasm. We only wish to give our views about the "real handy" and often almost indispensable terms, "vitality." Understood in this way, it seems to us to approach already somewhat nearer a clear conscious than when it is used with the vague notion of a self-conception entity, "vital force," something of a homunculus within the homo.—*New England Journal of Dentistry.*

